# Constrained Optimization for Decision Making in Health Care Using Python: A Tutorial

**DOI:** 10.1177/0272989X231188027

**Published:** 2023-07-22

**Authors:** K. H. Benjamin Leung, Nasrin Yousefi, Timothy C. Y. Chan, Ahmed M. Bayoumi

**Affiliations:** Department of Mechanical and Industrial Engineering, University of Toronto, Toronto, ON, Canada; Scottish Ambulance Service, Edinburgh, Scotland, UK; Smith School of Business, Queen’s University, Kingston, ON, Canada; Department of Mechanical and Industrial Engineering, University of Toronto, Toronto, ON, Canada; MAP Centre for Urban Health Solutions, St. Michael’s Hospital, Unity Health Toronto, Toronto, ON, Canada; Department of Medicine, University of Toronto, Toronto, ON, Canada; Division of General Internal Medicine, University of Toronto, Toronto, ON, Canada; Institute of Health Policy, Management and Evaluation, University of Toronto, ON, Canada

**Keywords:** constrained optimization, Python tutorial, prescriptive analytics, resource allocation

## Abstract

Constrained optimization can be used to make decisions aimed at maximizing some quantity in the face of fixed limits, such as resource allocation problems in health where tradeoffs between alternatives are inherent, and has been applied in a variety of health-related applications. This tutorial guides the reader through the process of mathematically formulating a constrained optimization problem, providing intuitive explanations for each component within the problem. We discuss how constrained optimization problems can be implemented using software and provide instructions on how to set up a solution environment using Python and the Gurobi solver engine. We present 2 examples from the existing literature that illustrate different constrained optimization problems in health and provide the reader with Python code used to solve these problems as well as a discussion of results and sensitivity analyses. This tutorial can be used to help readers formulate constrained optimization problems in their own application domains.

**Highlights:**

- This tutorial provides a user-friendly guide to mathematically formulating constrained optimization problems and implementing them using Python.
- Two examples are presented to illustrate how constrained optimization is used in health applications, with accompanying Python code provided.

Many decision-making problems in health involve attempts to maximize some quantity in the setting of fixed constraints. For example, a physician may want to select an antibiotic that maximizes the probability a patient will be cured of an infection while adhering to antibiotic stewardship guidelines that limit the use of broad-spectrum antibiotics to minimize the risk of antibiotic resistance. The manager of a diagnostic imaging department may wish to minimize wait times for a magnetic resonance imaging scan for patients with the highest likelihood of having severe illness constrained by the number of schedule times available. A minister of health may have to decide on the optimal mix of inpatient and outpatient services in a health system within a fixed budget.

Each of these examples represents a tradeoff. Constrained optimization is a mathematical method for determining the optimal solution to such decisions when the factors in the tradeoff can be quantified. More specifically, it is a systematic approach to finding the optimum (minimum or maximum) of all possible solutions for a set of decision choices that are subject to well-defined preconditions. Constrained optimization is a key component within prescriptive analytics, which aims to use data to improve decision making, and has been used in a variety of health-related applications.

While previous articles have reviewed the principles and potential use cases of constrained optimization within health,^1–4^ we believe that there is need for a user-friendly, introductory tutorial to optimization. In this tutorial, we present an overview of optimization with 2 worked examples: one of investment in HIV programs and another of placement of public defibrillators. We selected these examples as they are representative of 2 different and realistic decision-making problems encountered in many health care contexts. In addition to formulating and solving optimization models for each example, we present extensions by adding uncertainty and equity considerations. Each example includes a detailed explanation and annotated code that can be run by the user. All of the code and ancillary files are provided at (https://www.github.com/nyousefi2020/MDM-Tutorial).

## Methods

In this section, we first provide an overview of the components of a general mathematical optimization model. Next, we review some software for coding and solving such models. Finally, we explore, in detail, 2 worked examples of optimization modeling in medical decision-making applications. For these 2 applications, we provide code in the Python programming language as well as annotated model outputs.

### Overall Structure of an Optimization Model

An optimization model is characterized by 4 main features: 1) decision variables, 2) parameters, 3) an objective function, and 4) constraints.

#### Decision variables

Decision variables are the unknown quantities of an optimization model that are changed to achieve the desired outcome. For example, a decision variable could be the amount invested in a health care intervention. Values of the decision variables are varied systematically until an optimal solution is reached.

#### Parameters

Parameters are the input data for the problem. They are fixed numerical values that describe a particular instance of the problem that the model is aiming to solve. For example, the costs of specific interventions may be fixed parameters for a decision problem.

#### Constraints

Constraints dictate the allowable choices for the decision variables. A monetary budget is an example of a constraint, since it represents a limit on the total amount of funding that can be allocated across all strategies. In an optimization problem, decision variables cannot take on values that result in any constraint being violated. Constraints are written as equalities or inequalities using decision variables and parameters.

#### Objective function

The objective function is what the optimization model aims to minimize or maximize. It is a function of the decision variables and parameters. For example, the objective could be to maximize the aggregate health of a population. The objective function and constraints typically reflect a synergistic “tension.” For example, in a resource allocation problem, the objective function may aim to increase the population’s health while the constraint on the budget ensures that the costs are within a specified limit. Without this constraint, the model would suggest funding all the potential interventions. Similarly, without the objective function, it would not be possible to differentiate between decisions that have higher or lower health benefits.

#### Visual summary

Putting the 4 components together, a general optimization model can be formulated as follows:

$$\begin{array}{c} \mathrm{maximize}\;f\left(x_{1},\ldots ,x_{n};\alpha_{1},\ldots ,\alpha_{k}\right)\\ \mathrm{subject}\;\mathrm{to}\;g_{i}\left(x_{1},\ldots ,x_{n};\alpha_{1},\ldots ,\alpha_{k}\right)\geq 0,\;i=1,\ldots ,m \end{array}$$

This optimization model aims to maximize an objective function f with n decision variables $x_{1},\ldots ,x_{n}$ and k parameters $\alpha_{1},\ldots ,\alpha_{k}$ . The set of constraints $g_{i}$ limits the choices of the decision variables and is also a function of the decision variables and the parameters. Note that this model is general, meaning any optimization problem can be represented in this way. For example, a minimization problem can be transformed to this form by negating the objective function, or an equality constraint can be written as 2 inequality constraints.

The above model can be further specified along several dimensions. One important dimension pertains to whether the decision variables can take values in a continuous range or only discrete values in a set. A common example of a continuous optimization model is a linear program, in which the variables are continuous and the objective and constraints are linear functions of the decision variables:

$$\begin{array}{c} \mathrm{maximize}\;c_{1}x_{1}+\ldots +c_{n}x_{n}\\ \mathrm{subject}\;\mathrm{to}\;a_{i1}x_{1}+\ldots +a_{\mathrm{in}}x_{n}\geq b_{i},\;i=1,\ldots ,m \end{array}$$

The parameters $c_{1},\ldots ,c_{n}$ are objective function coefficients, $b_{i}$ is the right-hand-side parameter that governs constraint i , and $a_{i1},\ldots ,a_{\mathrm{in}}$ are constraint coefficients that affect constraint i .

In discrete optimization, all or some of the variables take values in a discrete set (e.g., binary or integer values). These optimization problems are referred to as integer programs or mixed-integer programs, respectively. Analogous to the continuous case, if the objective and constraints are linear, then the models are integer linear programs or mixed-integer linear programs.

### Software

Real-life optimization models require the use of computer software to solve and identify optimal solutions. Software used in optimization models can be classified into 2 groups: solver software (which we will call a “solver”) and modeling software. A solver takes an instance of an optimization model as an input and applies an algorithm to find an optimal solution. It is the “engine” that solves the problem. Modeling software connects a human modeler and solver by providing an environment to import data, generate model instances, call solvers, and analyze data. It is the “language” that allows an optimization problem to be represented and understood by the computer.

In general, a solver can be used in many different modeling languages. Similarly, modeling languages can connect to many different solvers. Some solvers have their own integrated modeling system. The ability of a solver to be embedded into a modeling system (e.g., an object-oriented programming language) is an important factor in selecting the solver. Commercial solvers such as CPLEX and Gurobi are most commonly used in industry and academia. Using these solvers requires purchasing a subscription, but free licenses are available for academics. Open-source solvers are also available. In this tutorial, we use Python as the modeling language and Gurobi as the solver.

Python is a powerful open-source programming language that is easy to learn for both beginner programmers and those who have experience with other languages. Moreover, its ready-made libraries and modules provide a lot of flexibility for programmers. For these reasons, it has become one of the most popular programming languages in the world. Python is a great choice for constrained optimization because of its advanced mathematical and scientific computing tools.

Gurobi is a fast and efficient optimization solver that can solve all major optimization problem types. It supports interfaces for a variety of programming and modeling languages including Python, C, C++, Java, MATLAB, and R. In this tutorial, we use Gurobi’s Python extension, “gurobipy,” to formulate and solve the optimization models.

### Setting up Anaconda and Gurobi

In this tutorial, we use Jupyter Notebook, a Web application that allows us to write and share Python code. The simplest way to install Python and Jupyter Notebook is through Anaconda, a popular distribution platform for data science and scientific computing. Anaconda Individual Edition is free and can be downloaded from the Anaconda Web site; we refer the reader to Anaconda’s installation guide for setup instructions.^5^

Next, users need to download and install the Gurobi optimization solver; installation instructions can be found on Gurobi’s Web site.^6^ The use of Gurobi requires an active license, which is free for academic users and has a limited-time trial for all other users.^7^

Finally, users need to install the “gurobipy” extension within Anaconda so that the code written in Jupyter Notebook can successfully interface with the Gurobi solver. We refer the reader to Gurobi’s documentation for step-by-step installation instructions.^8^

Once Anaconda, Gurobi, and “gurobipy” have been successfully installed, users can open Jupyter Notebook by first opening the Anaconda Navigator application and then opening the Jupyter Notebook application from the Anaconda Navigator dashboard. This will launch a Web browser tab from which the user can create and access Jupyter Notebook files, which are in .ipynb format and contain a combination of code, text, and visualizations.

## Two Examples of Optimization Models

In this section, we present 2 case studies from the literature in which optimization was applied to a resource allocation decision in health care.

### Example 1: HIV Program Selection

An estimated 2.3 million people annually are infected with the human immunodeficiency virus (HIV).^9^ Publicly funded HIV programs may include antiretroviral therapy for people who are HIV positive,^10–13^ preexposure prophylaxis to prevent new HIV infections among people at risk, and community education.^14–16^ Decision makers may want to determine how to allocate resources between these strategies, which have very different costs and effects. Accordingly, the optimal combination of strategies for a given population may be unclear.

The goal of the optimization model is to determine how to allocate a global budget across HIV programs such that the overall benefit to the population across all programs is maximized while respecting minimum and maximum investment limits in each program. Below, we present an example based on the simplest linear programming formulation presented by Juusola and Brandeau,^17^ in which they aimed to maximize the number of quality-adjusted life-years (QALYs) gained through the investment in 3 HIV programs for men who have sex with men (for this example, we ignore time horizons and discount rates). This problem is conceptualized as the number of people that can be reached within each of 3 programs: antiretroviral therapy scale-up (ART), preexposure prophylaxis (PrEP), and community-based education (CBE).

This model assumes that programs are independent (that is, investment in one program does not change the potential gains from investment in an alternative program), each program can be scaled up or down linearly in terms of costs accrued or QALYs gained, and each program has both a lower bound (investment cannot be lower than a certain level) and upper bound (investment cannot exceed a certain level). In the mathematical formulation below, the index i takes values of 0, 1, or 2 depending on which program is being considered. This is done to match the index notation in Python, in which the first element of a list has an index of 0.

Decision variables:

$x_{i}$ : the number of people reached with program i parameters

$x_{i}^{\min}$ : the number of people reached with the minimum investment in program i

$x_{i}^{\max}$ : the number of people reached with the maximum investment in program i

$a_{i}$ : the number of QALYs gained for each person reached with program i

$c_{i}$ : the cost per person reached with program i

B : the global budget for HIV programs

Objective: to maximize the total number of QALYs gained by investing in the 3 available programs.

$$\max\sum_{i=0}^{2}a_{i}x_{i}$$

The total QALYs gained are the sum of the number of QALYs gained for each person multiplied by the number of people reached with each program.

Constraints:

$$\begin{array}{c} x_{i}^{\min}\leq x_{i}\leq x_{i}^{\max},\;i=0,1,2\\ \sum_{i=0}^{2}c_{i}x_{i}=B \end{array}$$

The first constraint sets a minimum and maximum level for the number of people who can be reached with each program. The second constraint ensures that the total monetary cost of all 3 investments is equal to the global budget.

We use the same set of programs as Juusola and Brandeau^17^: ART, PrEP, and CBE, and the same global budget of $10 billion. Table 1 summarizes the other parameter values.

**Table 1:** Parameter Values for HIV Programs

Parameter	Program 0 (ART)	Program 1 (PrEP)	Program 2 (CBE)
$x_{i}^{\min}$ (Minimum investment)	50,000	10,000	10,000
$x_{i}^{\max}$ (Maximum investment)	400,000	3,000,000	3,000,000
$a_{i}$ (QALYs per person reached)	15.53	1.24	0.222
$c_{i}$ (Cost per person reached)	118,100	140,393	272

QALY, quality-adjusted life-year.

Below, we provide a step-by-step guide for implementing the above problem using Python and Gurobi; note that we present costs in thousands. The code for this example is also available as a standalone file as “Example1.ipynb.” Comments in Python are denoted by the pound symbol (#). Note also that Python uses indentation to denote a block of code (such as a for loop). Improper use of indentation will return an error.

#### Code for example 1

# Import Gurobi packages

import gurobipy as gp

from gurobipy import GRB

# Create a Gurobi model object

m = gp.Model(‘HIV’)

# DECISION VARIABLES

# Create continuous variables

# indicating the number of people reached by each of 3 programs

x = m.addVars(3,vtype=GRB.CONTINUOUS,name="Program")

# PARAMETERS

# costs, per person reached, by each of 3 types of programs

# Cost values are divided by 1000 for computational reasons

c = [118.100,140.393,0.272]

#total cost

cost = x[0]*c[0]+x[1]*c[1]+x[2]*c[2]

# QALYs gained, per person reached, by each of 3 programs

a = [15.53,1.24,0.222]

# total qalys

qalys = x[0]*a[0]+x[1]*a[1]+x[2]*a[2]

# Budget

# The budget is divided by 1000 for computational reasons

B = 10000000.000

# CONSTRAINTS

# Budget

m.addConstr(cost==B)

# Limits

x[0].lb=50000

x[0].ub=400000

x[1].lb=10000

x[1].ub=3000000

x[2].lb=10000

x[2].ub=3000000

# OBJECTIVE

# Maximize the total number of QALYs gained

m.setObjective(qalys, GRB.MAXIMIZE)

# Run the optimization model

m.optimize()

# Print the optimal solution and its objective value

for v in m.getVars():

print("%s = %.4f" % (v.varName, v.x))

print("Max QALYs = %.4f" % m.objVal)

The first 2 lines import the Gurobi functions and classes that we will need for the optimization model. Python allows us to bind a module to a name in the current scope (the running file). Thus, when we use the prefix “gp,” Python knows that we are calling a function from the “gurobipy” package.

Next, we create a Gurobi model object. This object will hold each of the 4 model components that we outlined above. We can call our model by any name (or even leave it unnamed); here, we call the model “HIV.”

The decision variables are denoted by x. We specify that there are 3 decision variables and that they are continuous. In practice, the decision variables in this example, which refer to the number of people in each program, must take integer values. However, we set them as continuous for illustrative purposes. The most commonly used decision variable types are continuous (GRB.CONTINUOUS), integer (GRB.INTEGER), and binary (GRB.BINARY), with continuous being the default type. Note that the default numbering in Python starts at 0 rather than 1; thus, the programs are numbered 0 through 2 rather than 1 through 3. We have also given them the name “Program.”

Next, we define the parameters of the optimization model. We have defined the costs per person for each program by a vector called c (again, the first element is obtained by specifying c[0]). The total costs in the model will be calculated by multiplying the number of people in each program by the cost of each program and summing across all 3 programs. We similarly define the number of QALYs per person for each program by a vector called a. We also specify the global budget B.

Next, we add constraints to the model using the addConstr()function. In our example, we specify that the total cost should be equal to the global budget. We also define the lower (.lb) and upper (.ub) bounds for the decision variables.

We then use the setObjective() method to define the objective function. This method takes 2 arguments. The first argument is the objective function expression (in our example, qalys), and the second argument states whether the goal is minimization (GRB.MINIMIZE) or maximization (GRB.MAXIMIZE). Since the problem is one of maximization, we use GRB.MAXIMIZE.

Now that the model is fully built, we solve it using the optimize()function.

Gurobi automatically chooses the algorithm based on the model type and outputs some built-in attributes, such as the status of the optimization, the solution, the run time, and other information (not shown here). If the model is successfully solved, the optimal values of the variables and the objective value can be obtained, as in the last 3 lines, where we print the number of people reached by each program as well as the optimal objective value (the maximum number of QALYs gained).

#### Results

The model output from our print commands yields the following:

Program[0] = 65876.9687

Program[1] = 10000.0000

Program[2] = 3000000.0000

Max QALYs = 1701469.3235

Thus, the model indicates that Program[0], which is ART, should be funded to reach 65,877 people, Program[1], PrEP, should be funded to reach 10,000 people (which is the minimum allocation), and Program[2], CBE, should be funded to reach 3,000,000 people (which is the maximum allocation). The total QALYs gained are 1.70 million; no other allocation across these 3 programs will yield a greater number of QALYs.

**Figure 1:**
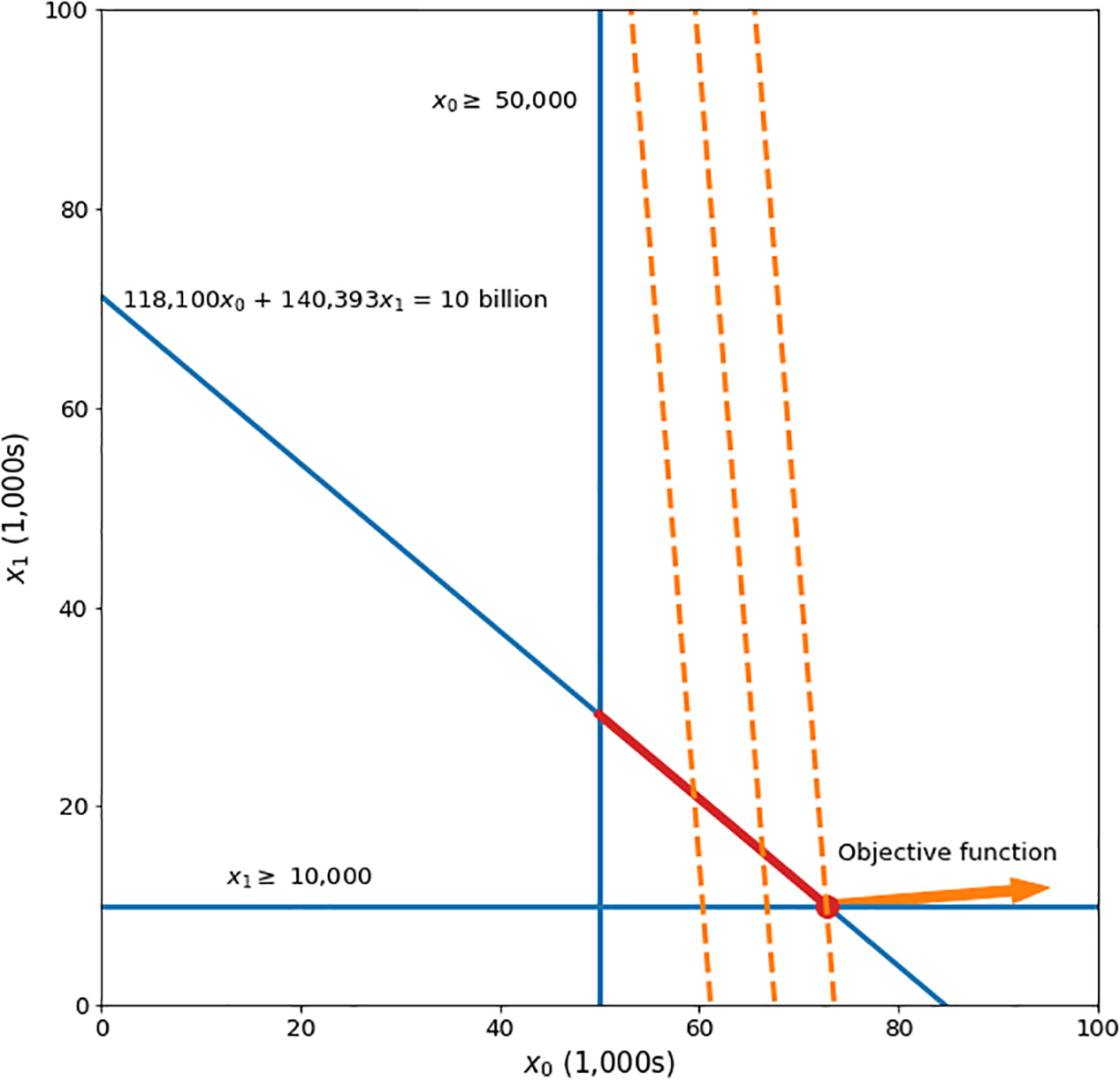
Graphical representation of the resource allocation problem for HIV programs when only considering Program 0 (ART) and Program 1 (PrEP).

To see if this result makes intuitive sense, we first consider only 2 programs: ART and PrEP. Figure 1 shows the graphical representation of the resulting optimization model. The blue lines are the lower bound and budget constraints. The upper bounds are far away to the right and do not affect the allowable choices of decision variables, so they are not shown in this figure. The red line represents the area that decision variables can be chosen from. This area is the intersection of the budget constraint and the lower and upper bound constraints. The orange lines are the level sets of the objective function. Optimality is reached at the intersection of the budget constraint and the lower bound constraint for decision variable $x_{1}$ .

Solving the model in Python confirms the conclusion from our visual inspection. In particular, the optimal solution is to invest the minimum amount required on PrEP ( $x_{1}$ = 10,000) and invest the remaining amount on ART ( $x_{0}$ = 72,786.37). This intuitively makes sense as ART has less cost and more per-person benefit. The optimal strategy results in 1.14 million QALYs.

Now, we consider all 3 programs, where we obtain the optimal solution using the Python code above. The optimal solution is to invest the minimum amount required on PrEP ( $x_{1}$ = 10,000) and maximum possible amount on CBE ( $x_{2}$ = 3,000,000) and invest the remaining amount on ART ( $x_{0}$ = 65,877). This intuitively makes sense as CBE has the highest relative benefit while PrEP has the lowest relative benefit. The optimal strategy results in 1.70 million QALYs.

**Figure 2:**
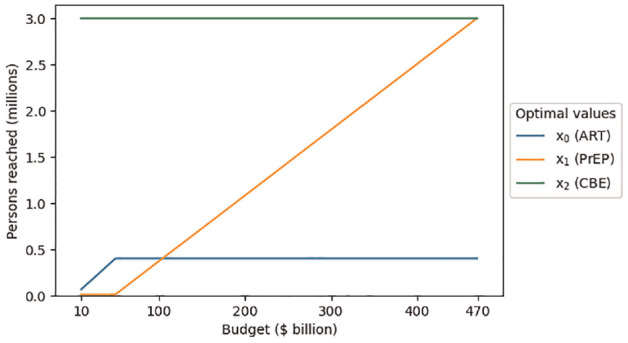
Optimal decision variable values as a function of adjusting the budget parameter.

We further examine the effect of the budget on the optimal strategy. Figure 2 shows the effect of increasing the budget on the optimal decision variables. The first point of each decision variable is its optimal solution from the original problem where the budget is $10 billion.

The variable for the third program (CBE) is already at its maximum (3,000,000), so increasing the budget does not allow for more participants to be added to that program. The variable for the first program (ART) reaches its maximum (400,000) when the budget increases to $49.5 billion. The variable for the second program (PrEP) then constantly increases as the budget increases until it reaches its maximum (3,000,000) at a budget of $469.2 billion. Further increasing the budget would have no effect since all 3 programs are already at their maximum limit.

#### Rewriting the code using matrices and arrays

A common approach in constrained optimization is using matrix notation to simplify the model and write it in a compact form, which is especially useful when the model has many decision variables and/or constraints.

The code for example 1 can be rewritten as follows. It is also available as a standalone file as ‘Example1_matrixnotation.ipynb’:

# Import necessary packages

import gurobipy as gp

from gurobipy import GRB

import numpy as np

# PARAMETERS

# costs, per person reached, by each of 3 types of programs

# Cost values are divided by 1000 for computational reasons

c = np.array([118.100,140.393,0.272])

# QALYs gained, per person reached, by each of 3 programs

a = np.array([15.53,1.24,0.222])

# Minimum/maximum number of persons reached by each program

# in array form

x_min = np.array([50000,10000,10000])

x_max = np.array([400000,3000000,3000000])

# Budget

# The budget is divided by 1000 for computational reasons

B = 10000000.000

# Create a Gurobi model

m = gp.Model(‘HIV’)

# DECISION VARIABLES

x = m.addMVar(3, lb=x_min, ub=x_max, vtype=GRB.CONTINUOUS, name="Program")

# CONSTRAINT

# The total cost across all programs must equal the budget

m.addConstr(c@x == B)

# OBJECTIVE

# Maximize the total number of QALYs gained

m.setObjective(a@x, GRB.MAXIMIZE)

# Run the optimization model

m.optimize()

# Print the optimal solution and its objective value

for v in m.getVars():

print("%s = %.4f" % (v.varName, v.x))

print("Max QALYs = %.4f" % m.objVal)

First, we import the gurobipy package, as well as the NumPy package, which will enable us to efficiently manipulate Python arrays in the model. As NumPy is already included within the Anaconda distribution, there is no need to install it separately.

We then define arrays for the cost parameters (c), the objective function coefficients (a), the lower bounds (x_min), and the upper bounds (x_max). The right-hand-side of the budget constraint (B) is a scalar parameter.

Creating the model object is the same as before. We define the decision variables in matrix form using addMVar()method. In this setting, we also use lower bound (lb) and upper bound (ub) arguments to define the bounds for the decision variables so that we do not need to add them as constraints. The default for lower bound is 0 and for upper bound is infinite (GRB.INFINITY). For the decision variables x, the lower and upper bounds are x_min and x_max, respectively.

Finally, we add the budget constraint, and the rest of the model is similar to the previous code. Note that the operator @ is used for matrix or vector multiplications.

#### Incorporating parameter uncertainty

The above formulation assumes that the parameter values are known with certainty. In practice, many parameter values are uncertain, which we address through an extension to our linear programming formulation using a technique called *stochastic programming*.

Suppose that the number of QALYs gained for each person reached with each program is uncertain. To address this uncertainty, relevant parameter values are estimated using subjective elicitation from experts, related literature, and past data. Each combination of parameter values forms a scenario. In addition to estimating parameter values, we also incorporate the probability of each scenario being realized (using similar data sources).

In this example, we assume that there are 3 scenarios (sets of estimates), yielding 3 estimates of the number of QALYs gained per person with each program. Table 2 presents the parameter values for each scenario and the probability that each scenario will occur. These values were chosen purely for illustrative purposes. The cost and budget parameters remain unchanged from the previous example.

**Table 2:** Estimates of the Number of QALYs Gained per Person Reached for Each Program in Each Scenario

Scenario ( j )	Probability of Ccenario ( $p_{j}$ )	QALYs per Person Reached $\left(a_{i}\right)$		
		Program 0 (ART)	Program 1 (PrEP)	Program 2 (CBE)
0	0.4	3.06	20.48	0.05
1	0.5	15.53	1.24	0.222
2	0.1	2.42	40.60	0.02

QALY, quality-adjusted life-year.

Optimizing the expected objective value is a common approach to finding an optimal solution for a problem with uncertain parameters. A naïve way of doing this is to try every possible solution and calculate the expected objective value over all the scenarios. However, this is time-consuming and unlikely to be practical. Instead, we can use stochastic programming to solve a single problem instance, which consists of finding a solution that maximizes the expected objective function value, calculated as the number of people reached with each program in each scenario multiplied by the probability of that scenario occurring, summed over all scenarios:

$$\max\sum_{j=0}^{2}p_{j}\sum_{i=0}^{2}a_{\mathrm{ij}}x_{i}$$

To implement this change, we modify the part of the code that defines the number of QALYs gained for each person reached with each program and add in the probability of each scenario:

# QALYs gained, per person reached, by each program

# in each of 3 scenarios

a_scenario1 = np.array([3.06,20.48,0.05])

a_scenario2 = np.array([15.53,1.24,0.222])

a_scenario3 = np.array([2.42,40.60,0.02])

# Compute the expected number of QALYs gained per person reached

a = 0.4*a_scenario1 + 0.5*a_scenario2 + 0.1*a_scenario3

Note that the last line calculates the expected value of the parameters over all scenarios, so we do not need to change the objective function part in the code. The complete code that includes this extension is available in the file named “Example1_stochastic.ipynb.”

In this example, we restricted our problem to 3 scenarios for illustrative purposes. While incorporating a greater number of scenarios can lead to a more realistic model, doing so can rapidly increase the model’s size, which may lead to computational challenges.

The model output is:

Program[0] = 50000.0000

Program[1] = 23355.8653

Program[2] = 3000000.0000

Max QALYs = 1161186.6984

This output indicates that Program[0], ART, should be funded to reach 50,000 people (less than before); Program[1], PrEP, should be funded to reach 23,356 people (more than before); and Program[2], CBE, should be funded to reach 3,000,000 people (same as before), with the expected number of QALYs gained across all programs and scenarios equaling 1.16 million.

### Example 2: Public Defibrillator Placement

Out-of-hospital cardiac arrest affects more than 6 million adults annually worldwide. Of these, only 10% to 15% of victims survive to hospital discharge.^18–20^ If an electric shock delivered by an automated external defibrillator (AED) is applied to the victim before the arrival of emergency medical services, the odds of survival can increase by up to 3-fold.^21,22^ Survival is time sensitive: a longer delay between the onset of cardiac arrest and the application of an AED is negatively associated with likelihood of survival.^23^ Since the locations of cardiac arrests have been found to be spatially stable over time,^24,25^ constrained optimization can be used to determine the ideal locations for AEDs to maximize their proximity to historical (and future) cardiac arrest locations.

American Heart Association guidelines suggest that AEDs should be located in high-risk areas of cardiac arrest such that they can be retrieved and applied by laypeople to a cardiac arrest victim before the arrival of emergency medical services.^26^ Several studies have used the concept of “coverage” to measure AED effectiveness: a cardiac arrest that is within a certain distance from any AED is considered to be “covered,” meaning that an AED could potentially be used to respond to that cardiac arrest.^27–32^ A distance of 100 m is typically used as a cutoff for coverage to approximate a 3-min round-trip travel time to retrieve and deploy the AED.^27,28,30^

The goal of the constrained optimization model is to determine the locations of a fixed number of new AEDs such that the number of covered cardiac arrests is maximized, while considering each potential AED location’s effectiveness at covering cardiac arrests. Below, we show an integer linear program formulation presented by Chan et al.^27^ Our problem instance will use an artificially generated data set consisting of 500 cardiac arrests and 1,500 candidate AED locations.

The problem formulation is as follows.

Decision variables:

$x_{j}$ : whether or not to place an AED at candidate location j {1 = yes, 0 = no}

$y_{i}$ : whether or not cardiac arrest i is covered {1 = yes, 0 = no}

Parameters:

$a_{\mathrm{ij}}$ : whether or not cardiac arrest i would be covered by an AED that is placed at candidate location j {1 = yes, 0 = no}. We precompute the value of $a_{\mathrm{ij}}$ based on a user-defined cutoff distance for coverage.

K : the total number of AEDs to be placed

Objective: to maximize the total number of covered cardiac arrests

$$\max\sum_{i=0}^{499}y_{i}$$

Constraints:

$$\begin{array}{c} a_{\mathrm{ij}}x_{j}\geq y_{i},\;i=0,\ldots ,499\\ \sum_{j=0}^{1499}x_{j}=K\\ x_{j}\in \left\{0,1\right\},\;j=0,\ldots ,1499\\ y_{i}\in \left\{0,1\right\},\;i=0,\ldots ,499 \end{array}$$

The first constraint ensures that a cardiac arrest is counted as covered only if at least 1 location with an AED placed can cover the cardiac arrest. The second constraint ensures that exactly K AEDs are placed. Finally, the third and fourth constraints ensure that all decision variables are binary.

#### Code for example 2

In contrast to example 1, this example has many decision variables and constraints; thus, it is preferred to use matrix notation in the code. The code for this example is provided below. It is also available as a standalone file as “Example2.ipynb”:

# Import necessary packages

import gurobipy as gp

from gurobipy import GRB

import numpy as np

import pandas as pd

# Load the data set that contains distances between

# cardiac arrests and potential AED locations

covered = pd.read_csv(‘Example2_distanceMatrix.csv’, index_col=‘ID’)

# PARAMETERS

# Coverage cutoff limit

coverage_distance = 100

# Number of AEDs to be placed

K = 25

# Compute a_ij, which states whether each cardiac arrest is

# within the coverage distance of each potential AED location

covered = (covered <= coverage_distance).astype(int)

n_cases = covered.shape[0]

n_candidates = covered.shape[1]

A = pd.DataFrame.to_numpy(covered)

# Create a Gurobi model object

m = gp.Model(‘AED’)

# DECISION VARIABLES

# x is whether an AED is placed at potential AED location j

x = m.addMVar(n_candidates, vtype = GRB.BINARY, name="x")

# y is whether cardiac arrest i is covered by at least one AED

y = m.addMVar(n_cases, vtype = GRB.BINARY, name="y")

# CONSTRAINTS

# Cardiac arrest covered if at least one AED is

# placed within range

m.addConstr(A@x >= y)

# Total number of AEDs placed

m.addConstr(x.sum() == K)

# OBJECTIVE

# Maximize the number of covered cardiac arrests

m.setObjective(y.sum(), gp.GRB.MAXIMIZE)

# Run the optimization model

m.optimize()

# Print the optimal solution and its objective value

selected = []

for j in x.tolist():

if j.X > 0.5:

selected.append(j.index)

print(″Selected locations: ″, end=″″)

print(*selected, sep=‘, ’)

print(″Number of covered cardiac arrests: ″ + str(int(m.objVal)))

The first 4 lines import the required classes and libraries. Since the parameters of this example are stored in a file, we also load Pandas, a data analysis package for Python. As is the case for NumPy, Pandas is included within the Anaconda distribution, so we do not need to install it separately.

We then load the data. The data set comes as a .csv file named “Example2_distanceMatrix,” where the rows and columns correspond to the cardiac arrests and candidate locations, respectively. The first column is labeled “ID” to indicate the cardiac arrest IDs, and the remaining columns are labeled as the candidate locations (numbered 0 to 1,499 in this example). The cells contain the distance of each cardiac arrest from each candidate location. The data file must be located in the same directory as the Jupyter Notebook file. We use the read_csv() method from Pandas to load the data. read_csv() has several arguments, of which we use only the file path/name (the first argument) and the column index (the second argument). The index_col argument sets the “ID” column as the index so that it is excluded from the distance data. Pandas stores the data set as a DataFrame, which we call “covered.”

Next, we set our parameters, which are the number of AEDs placed and the coverage limit.

Given the distances and the coverage limit, the next line transforms the distances to 0/1 data to be used in the model so that a cell takes value of 1 if the distance is less than or equal to the specified coverage limit. Now, the “covered” DataFrame contains the $a_{\mathrm{ij}}$ values for each arrest-location pair (i,j) . We define the number of arrests (n_cases) and the number of candidates (n_candidates) based on the dimensions of the DataFrame so that if we receive another data set with different number of arrests and candidate locations, the code does not need to change. Finally, using the to_numpy() method from Pandas, we convert the DataFrame, which contains the $a_{\mathrm{ij}}$ parameters, to a matrix form and call it “A.” This step is necessary as a DataFrame object cannot directly be used in the Gurobi model.

Now that the parameters are set, we create a Gurobi model object and call it “AED.”

Then, we add the decision variables x and y with their corresponding dimensions entered as the first argument. Since we set the variable type to binary (GRB.BINARY), we do not need to additionally define variable lower or upper bounds.

Next, we add the 2 constraints and set the objective function.

Finally, we solve the optimization model and print the optimal values of the decision variables and the objective function value.

#### Results

The model output from the above code is:

Selected locations: 1234, 1235, 1236, 1238, 1240, 1242, 1245, 1274, 1276, 1277, 1279, 1280, 1282, 1284, 1317, 1318, 1320, 1321, 1322, 1323, 1364, 1366, 1368, 1371, 1414

Number of covered cardiac arrests: 155

This output shows the indices of the K locations selected for AED placement and the total number of cardiac arrests that the selected locations cover.

**Figure 3:**
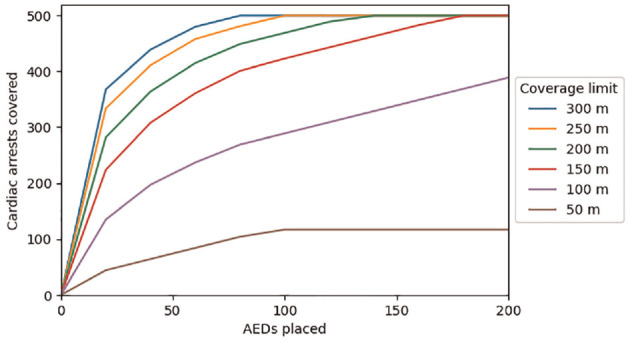
Cardiac arrest coverage as a function of the number of automated external defibrillators (AEDs) placed and the coverage distance.

We solved the resulting problem using different quantities of AEDs placed (parameter K ) and different coverage limits. The value of the objective function, which represents the total number of covered cardiac arrests, as a function of the number of AEDs placed and the coverage limit is shown in Figure 3.

As the number of AEDs placed increases, the total number of covered cardiac arrests increases until all 500 cardiac arrests in the data set are covered. However, the number of cardiac arrests covered by each additional AED placed diminishes as the total number of AEDs increases. For example, assuming a 200 m coverage limit, 4 AEDs are required to cover at least 100 cardiac arrests, 16 AEDs are required to cover at least 250 cardiac arrests, and 131 AEDs are required to cover all 500 cardiac arrests. This is because the optimization model will first place AEDs in locations that cover the greatest number of cardiac arrests, but subsequent AEDs will be placed in locations that cover fewer cardiac arrests.

The coverage limit also plays a role in determining the number of AEDs needed: given a fixed number of cardiac arrests to be covered, larger coverage limits result in a smaller number of AEDs required, while smaller coverage limits result in a larger number of AEDs required.

For coverage limits of 50 m and 100 m, the number of covered cardiac arrests output by the optimization model does not equal the total number of cardiac arrests in the data set, regardless of the number of AEDs placed. This is because for some cardiac arrests, the nearest candidate AED location is farther away than the coverage limit, meaning that none of the candidate AED locations would be able to cover that cardiac arrest.

#### Incorporating equity

Decision makers may want to find solutions that are not only efficient but also equitable. However, equity is complex, since it can be conceptualized in many different ways (e.g., equity of opportunity v. equity of access v. equity of outcome; horizontal v. vertical equity)^33,34^ and is often considered to be multidimensional (e.g., equity by geography, population, and health condition). Some of these equity considerations can be readily incorporated into a constrained optimization model through the objective function or constraints. Other approaches to incorporating equity in constrained optimization models are beyond the scope of this tutorial. Below, we provide a simple illustrative example that addresses geographic equity, based on a similar approach used by Leung et al.^35^

Suppose the study setting for our AED example is divided into 4 equally sized regions, each containing 375 candidate locations. For simplicity, let region 0 encompass candidate locations 0 through 374, region 1 encompass candidate locations 375 through 749, and so on. We introduce additional constraints that require each region to receive at least a proportion s of the total AEDs available, where s is a user-defined parameter. These constraints ensure that each region receives a minimum number of AEDs regardless of how many cardiac arrests occurred in that region:

$$\begin{array}{c} \sum_{j=0}^{374}x_{j}\geq \mathrm{sK}\\ \sum_{j=375}^{749}x_{j}\geq \mathrm{sK}\\ \sum_{j=750}^{1124}x_{j}\geq \mathrm{sK}\\ \sum_{j=1125}^{1499}x_{j}\geq \mathrm{sK} \end{array}$$

These constraints can be added to our code as shown below. The complete code that includes this extension can be found in the “Example2_equity.ipynb” file.

# Equity constraints

# Number of regions

n_regions = 4

# Minimum proportion of AEDs per region

s = 0.2

# Determine the number of candidate AED locations per region,

# assuming that each region has the same number of AEDs

break_point = int(n_candidates/n_regions)

# For each region, add a constraint that forces the number

# of AEDs placed in that region to be at least proportion s

# times the total number of AEDs available

for i in range(n_regions):

m.addConstr(x[break_point*i:(break_point*(i+1))].sum() >= (s*K))

The first 2 lines (ignoring comments) set the number of regions to 4 and the minimum proportion of AEDs per region to 0.2, respectively. Next, break_point is calculated as the number of candidate AED locations per region rounded up to the nearest integer. Finally, a for loop is used to add a constraint for each region that forces the number of AEDs placed in that region to be at least the proportion *s* times the total number of AEDs available (K). Note that x[break_point*i:(break_point*(i+1))].sum() represents the sum of candidate AED locations for region i.

The model output is as follows:

Selected locations: 98, 121, 139, 222, 265, 411, 645, 689, 694, 736, 779, 811, 813, 816, 821, 1234, 1235, 1236, 1276, 1280, 1282, 1318, 1320, 1322, 1323

Number of covered cardiac arrests: 116

The previous model, which did not incorporate equity constraints, resulted in covering 155 cardiac arrests. In comparison, enforcing that each region must receive at least 20% of the 25 AEDs (i.e., at least 5 AEDs per region) resulted in covering 116 cardiac arrests, a 25% decrease in coverage. However, the variation in the number of AEDs placed per region has decreased as well. In the model without the equity constraints, 1 region (region 3) receives all the AEDs, while the other 3 regions receive none. After adding the equity constraints, region 3 receives 10 AEDs, and the other regions receive 5 AEDs (total of *K* = 25 AEDs).

**Figure 4:**
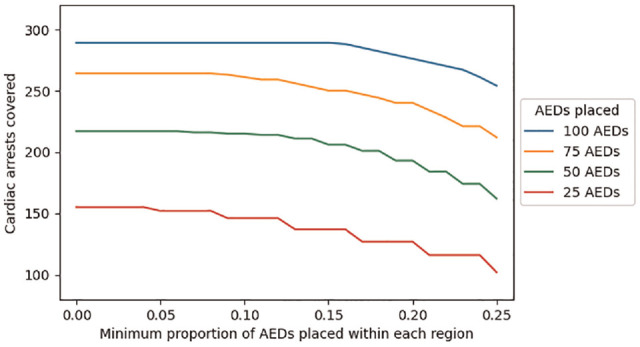
Cardiac arrest coverage as a function of the equity constraint parameter (*s*) and the number of automated external defibrillators (AEDs) placed.

**Figure 5:**
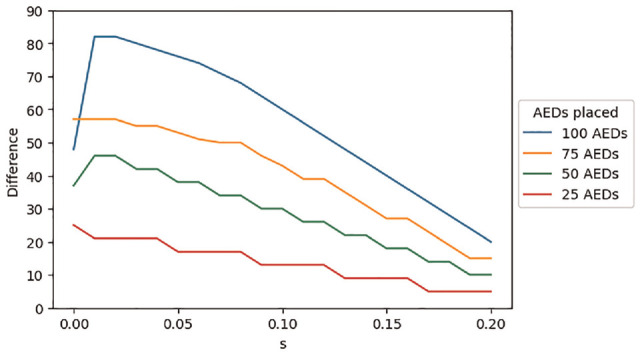
Difference in the number of automated external defibrillators (AEDs) placed between the region with the most AEDs and the region with the fewest AEDs, as a function of the equity constraint parameter (*s*) and the total number of AEDs placed across all 4 regions.

The total number of covered cardiac arrests and the difference between the regions getting the most and least AEDs, as a function of the constraint parameter s and the number of AEDs placed, are shown in Figures 4 and 5, respectively. As each region receives a higher proportion of AEDs (i.e., as *s* increases), the difference between the regions getting the most and least AEDs decreases, which may be viewed as an equity gain, while the total number of covered cardiac arrests decreases, which may be viewed as an efficiency loss. Accordingly, this example illustrates how constrained optimization can be used to explore equity-efficiency tradeoffs, which are intrinsic to many resource allocation decisions.

## Discussion

We have provided an overview of how to implement constrained optimization with 2 health-related examples. Although our examples were stylized for ease of exposition, the level of model complexity is comparable to many published examples. Readers of this tutorial can readily apply these principles to real-world problems.

Constrained optimization has the potential to extend methods commonly used by decision scientists in health care. For example, cost-effectiveness analyses typically assume that programs are perfectly divisible and associated with constant returns to scale (the ratio of costs to effects is constant, even if a program is partially implemented), but these assumptions may be unrealistic in many real-world contexts. Constrained optimization can address such limitations and consider decisions where programs are either not divisible or can only be divided in certain configurations or where returns to scale are a function of program size. Furthermore, decision makers often need to consider other factors in the objective beyond cost and health effects, which can be easily incorporated into constrained optimization models.

Constrained optimization problems may be challenging to implement for several reasons.

First, each parameter of the decision problem needs to be known and quantified, which may be challenging when this information is not readily accessible. We have provided an example of using stochastic programming when complete knowledge about the values of parameters is not available. However, stochastic programming is only one of several techniques that are used for decision making under uncertainty. Alternative methods include probabilistic sensitivity analysis (simulations in which uncertain parameters are repeatedly drawn from probability distributions) and robust optimization (decisions are optimized against worst-case scenarios). These methods are described in detail in advanced texts.^36,37^

Second, the objective function needs to be well defined. While decision problems can have several objectives, they can have only a single objective function. Practical decision problems with multiple objectives will need to combine these objectives into one by weighting and aggregating or by using a hierarchical order for objectives.

Third, solvers may not find optimal solutions for some decision problems. Solver issues may be related to specific algorithms that a solver is using (which may be addressed by using alternative algorithms) or, more commonly, because the problem specified in such a way that makes it mathematically infeasible or is too large to solve to optimality. In the latter case, solvers may find approximate solutions, which may still be useful for decision making.

In summary, constrained optimization is an important addition to the toolkit of decision scientists, and there are many types of problems that can be considered within a constrained optimization framework. Optimization software is readily available at low or no cost for academic researchers and can be integrated with commonly used programming languages such as Python and R. We believe that constrained optimization has been underused in health and hope that this guidance helps to increase its adoption.

## Supplemental Material

sj-csv-4-mdm-10.1177_0272989X231188027 – Supplemental material for Constrained Optimization for Decision Making in Health Care Using Python: A Tutorial Click here for additional data file. Supplemental material, sj-csv-4-mdm-10.1177_0272989X231188027 for Constrained Optimization for Decision Making in Health Care Using Python: A Tutorial by K. H. Benjamin Leung, Nasrin Yousefi, Timothy C. Y. Chan and Ahmed M. Bayoumi in Medical Decision Making

sj-ipynb-1-mdm-10.1177_0272989X231188027 – Supplemental material for Constrained Optimization for Decision Making in Health Care Using Python: A Tutorial Click here for additional data file. Supplemental material, sj-ipynb-1-mdm-10.1177_0272989X231188027 for Constrained Optimization for Decision Making in Health Care Using Python: A Tutorial by K. H. Benjamin Leung, Nasrin Yousefi, Timothy C. Y. Chan and Ahmed M. Bayoumi in Medical Decision Making

sj-ipynb-2-mdm-10.1177_0272989X231188027 – Supplemental material for Constrained Optimization for Decision Making in Health Care Using Python: A Tutorial Click here for additional data file. Supplemental material, sj-ipynb-2-mdm-10.1177_0272989X231188027 for Constrained Optimization for Decision Making in Health Care Using Python: A Tutorial by K. H. Benjamin Leung, Nasrin Yousefi, Timothy C. Y. Chan and Ahmed M. Bayoumi in Medical Decision Making

sj-ipynb-3-mdm-10.1177_0272989X231188027 – Supplemental material for Constrained Optimization for Decision Making in Health Care Using Python: A Tutorial Click here for additional data file. Supplemental material, sj-ipynb-3-mdm-10.1177_0272989X231188027 for Constrained Optimization for Decision Making in Health Care Using Python: A Tutorial by K. H. Benjamin Leung, Nasrin Yousefi, Timothy C. Y. Chan and Ahmed M. Bayoumi in Medical Decision Making

sj-ipynb-5-mdm-10.1177_0272989X231188027 – Supplemental material for Constrained Optimization for Decision Making in Health Care Using Python: A Tutorial Click here for additional data file. Supplemental material, sj-ipynb-5-mdm-10.1177_0272989X231188027 for Constrained Optimization for Decision Making in Health Care Using Python: A Tutorial by K. H. Benjamin Leung, Nasrin Yousefi, Timothy C. Y. Chan and Ahmed M. Bayoumi in Medical Decision Making

sj-ipynb-6-mdm-10.1177_0272989X231188027 – Supplemental material for Constrained Optimization for Decision Making in Health Care Using Python: A Tutorial Click here for additional data file. Supplemental material, sj-ipynb-6-mdm-10.1177_0272989X231188027 for Constrained Optimization for Decision Making in Health Care Using Python: A Tutorial by K. H. Benjamin Leung, Nasrin Yousefi, Timothy C. Y. Chan and Ahmed M. Bayoumi in Medical Decision Making
